# Preliminary findings from the Delirium and Population Health Informatics Cohort (DELPHIC) – Qatar Study

**DOI:** 10.3126/nje.v14i2.69366

**Published:** 2024-09-02

**Authors:** Hanadi Al Hamad, Mani Chandran, Pravija Talapan Manikoth, Daniel Davis, Brijesh Sathian

**Affiliations:** 1 Geriatrics and long term care department, Rumailah Hospital, Hamad Medical Corporation, Doha, Qatar; 2 University College London Hospitals NHS Foundation Trust, London, UK

**To the Editor:**

The Delirium and Population Health Informatics Cohort (DELPHIC) -Qatar project is a prospective longitudinal population-based cohort study aimed at establishing the relationship between delirium and dementia in Qatar. Delirium is a common complication requiring hospitalization in people aged ≥ 70 years. The prevalence of dementia is also increasing in the elderly, and the symptoms of delirium and dementia are difficult to distinguish. Furthermore, delirium may increase the risk of dementia [1]. This project provides a unique opportunity to prospectively evaluate cognitive and functional changes and the association between delirium and dementia.

The DELPHIC-Qatar project will establish the Delirium Hub in Rumailah Hospital by recruiting 2000 patients aged ≥ 70 years from different disciplines within HMC. It provides a strong clinical and translational research platform for developing cross-disciplinary collaboration within HMC. The standardized procedures of screening, recruiting, consenting, and assessing patients for the project within the Delirium Hub will provide a basis for the development of high-quality translational research within the HMC and will also improve patient care.

The DELPHIC-Qatar cohort study enrolled a total of 638 participants aged 70 years and above, with 277 males and 361 females from 10^th^ March 2021 to 30^th^ June 2024. The sex-wise distribution of risk and outcomes revealed significant findings. Among the participants, 47.8% were classified as high risk, with a higher proportion of females (50.7%) than males (44.0%). Conversely, 34.6% of the cohort was categorized as low-risk, with 36.5% of men and 33.2% of women. Delirium was observed in 17.6% of the total cohort, with a slightly higher incidence in males (19.5%) than in females (16.1%). Mortality rates were comparable between the sexes, with 23.8% in males and 23.0% in females, resulting in an overall mortality rate of 23.4% in the cohort.

Bivariate logistic regression analysis indicated that patients with delirium had a significantly increased risk of mortality, with a threefold higher risk (COR 3, 95% CI: 1.9–4.6; p<0.01). These findings underscore the strong association between delirium and adverse outcomes, particularly mortality, highlighting the critical need for the early identification and management of delirium in the elderly population to mitigate its impact on long-term health outcomes.

According to the study by Gordon et al., patients with delirium had a 39% greater chance of mortality (hazard ratio 1.39, 95% confidence range 1.37 to 1.41) and a threefold higher risk of onset dementia (hazard ratio 3.00, 95% confidence interval 2.91 to 3.10) than those without delirium [2].

The DELPHIC-Qatar project is expected to make major advances in our knowledge of the complicated connection that exists between delirium and dementia in the elderly population. By building the Delirium Hub and utilizing a large, varied cohort, this project will give vital insights into the cognitive and functional pathways associated with delirium, as well as its possible role in the development of dementia. Standardized methods and cross-disciplinary collaboration within HMC will not only increase the quality of translational research, but will also result in better clinical practices and patient outcomes in Qatar. As the project develops, its findings will be critical in determining future methods for preventing, detecting, and managing delirium and dementia, eventually helping Qatar's aging population.

## Figures and Tables

**Table 1:** Gender wise comparison DELPHIC cohort

Gender	Male (n=277)	Female (n=361)	Total (n =638)
**High Risk**	122 (44.0%)	183 (50.7%)	305 (47.8%)
**Low Risk**	101 (36.5%)	120 (33.2%)	221 (34.6%)
**Delirium**	54 (19.5%)	58 (16.1%)	112 (17.6%)
**Mortality**	66 (23.8%)	83 (23.0%)	149 (23.4%)
